# Supplementary material for: FDA orphan drug designations for lysosomal storage disorders – a cross-sectional analysis
Source: PLoS One. 2020 Apr 8;15(4):e0230898. doi: 10.1371/journal.pone.0230898 (PMC7141691; doi:10.1371/journal.pone.0230898)
Supplement: Attachment — Submitted filename: response to reviewers.docx [file pone.0230898.s002.docx]

PONE-D-20-00658+

FDA orphan drug designations for lysosomal storage disorders - a cross sectional analysis

PLOS ONE

We thank the editor and the reviewers for their thoughtful comment which were very helpful to further strengthen your manuscript.

We are carefully addressing the issues point-by-point in the following paragraphs. The comments are numbered in chronological order.

**Journal Requirements:**

Comment 1: when submitting your revision, we need you to address these additional requirements:

1. Please ensure that your manuscript meets PLOS ONE's style requirements, including those for file naming. The PLOS ONE style templates can be found at http://www.plosone.org/attachments/PLOSOne_formatting_sample_main_body.pdf and http://www.plosone.org/attachments/PLOSOne_formatting_sample_title_authors_affiliations.pdf

Answer 1: We have adjusted the title page and the main text according to <https://journals.plos.org/plosone/s/file?id=ba62/PLOSOne_formatting_sample_title_authors_affiliations.pdf>

<https://journals.plos.org/plosone/s/file?id=80c1/PLOSOne_formatting_sample_main_body.pdf>

Changes are marked in yellow in the marked-up version.

Comment 2:

1. Thank you for stating the following in the Financial Disclosure section:

"The authors received no specific funding for this work."

We note that one or more of the authors are employed by a commercial company: SphinCS GmbH.

Please provide an amended Funding Statement declaring this commercial affiliation, as well as a statement regarding the Role of Funders in your study. If the funding organization did not play a role in the study design, data collection and analysis, decision to publish, or preparation of the manuscript and only provided financial support in the form of authors' salaries and/or research materials, please review your statements relating to the author contributions, and ensure you have specifically and accurately indicated the role(s) that these authors had in your study. You can update author roles in the Author Contributions section of the online submission form.

Please also include the following statement within your amended Funding Statement.

“The funder provided support in the form of salaries for authors [insert relevant initials], but did not have any additional role in the study design, data collection and analysis, decision to publish, or preparation of the manuscript. The specific roles of these authors are articulated in the ‘author contributions’ section.”

If your commercial affiliation did play a role in your study, please state and explain this role within your updated Funding Statement.

2. Please also provide an updated Competing Interests Statement declaring this commercial affiliation along with any other relevant declarations relating to employment, consultancy, patents, products in development, or marketed products, etc.

Within your Competing Interests Statement, please confirm that this commercial affiliation does not alter your adherence to all PLOS ONE policies on sharing data and materials by including the following statement: "This does not alter our adherence to PLOS ONE policies on sharing data and materials.” (as detailed online in our guide for authors http://journals.plos.org/plosone/s/competing-interests) . If this adherence statement is not accurate and there are restrictions on sharing of data and/or materials, please state these. Please note that we cannot proceed with consideration of your article until this information has been declared.

Please include both an updated Funding Statement and Competing Interests Statement in your cover letter. We will change the online submission form on your behalf.

Please know it is PLOS ONE policy for corresponding authors to declare, on behalf of all authors, all potential competing interests for the purposes of transparency. PLOS defines a competing interest as anything that interferes with, or could reasonably be perceived as interfering with, the full and objective presentation, peer review, editorial decision-making, or publication of research or non-research articles submitted to one of the journals. Competing interests can be financial or non-financial, professional, or personal. Competing interests can arise in relationship to an organization or another person. Please follow this link to our website for more details on competing interests: http://journals.plos.org/plosone/s/competing-interests

Answer 2: We have have amended the funding statement and conflict of interest statement for Dr. Mengel as follows in the cover letter:

Funding statement: SphinCS GmbH provided support in the form of salary for EM, but did not have any additional role in the study design, data collection and analysis, decision to publish, or preparation of the manuscript. The specific roles of this author are articulated in the ‘author contributions’ section.

Conflict of interest: EM is managing partner of SphinCS GmbH, a private clinical research institute, which had research grants from Sanofi Genzyme, Takeda, Alexion, Orphazyme and Idorsia. This does not alter our adherence to PLOS ONE policies on sharing data and materials.

Comment 3: Please include captions for your Supporting Information files at the end of your manuscript, and update any in-text citations to match accordingly. Please see our Supporting Information guidelines for more information: http://journals.plos.org/plosone/s/supporting-information.

Answer 3: We added a caption for the supporting information at the end of the manuscript and reference this supporting information in the methods section.

**Reviewers' comments:**

Reviewer #1:

Comment 4: The review explains the state of the art of development of treatments for LSDs. It is well written and with complete information.

The manuscript is well organized and clearly written.

I would suggest to change the figures, the points alone in them are difficult to understand. May be if you put a line, it could be better understandable.

Answer 3: we thank the reviewer for this encouraging comment. We have amended figures 2A, 3A, 2B, and 3B with lines and/or captions that render the figures more intuitively to read.

Reviewer #2:

Comment 5: The manuscript by Garbade et al, entitled "FDA orphan drug designations for lysosomal storage disorders - a cross sectional analysis" represents a useful summary and analysis of all orphan drug designation related to lysosomal storage disorders, compared to all orphan drug designations in the same period of time.

Although well-written, the manuscript presents a strange form, since Authors, instead of describing their results citing related Figures and Tables, as commonly performed, here present their results mainly using the Figure and Table legends, which renders results very difficult to read. Therefore, presentation of results should be totally re-written.

Comment 5: We agree that the PLOS ONE manuscript structure is different from many other journals.

We have rechecked the requirements on <https://journals.plos.org/plosone/s/submission-guidelines> :

1. Figures

“Do not include figures in the main manuscript file. Each figure must be prepared and submitted as an individual file”

2. Figure captions

“Figure captions must be inserted in the text of the manuscript, immediately following the paragraph in which the figure is first cited (read order). Do not include captions as part of the figure files themselves or submit them in a separate document.”

3. Tables

“Cite tables in ascending numeric order upon first appearance in the manuscript file.

Place each table in your manuscript file directly after the paragraph in which it is first cited (read order). Do not submit your tables in separate files.”

The presentation of figures and tables as it stands is therefore compliant with PLOS ONE’s format.

We re-wrote the results section completely. In order to avoid fragmentation of the text, we aggregated the word flow and created larger paragraphs. In addition, the figure titles and table titles are printed in bold now which makes it much easier to differentiate them from the main text.

Comment 6: In addition, Authors should provide all Figure and Table legends in a section separated from the main text. In its present form, the paper is really hard to understand and quite confusing.

Answer 6: We agree that this is common practice in many other journals. Changing this would not be compliant with PLOS ONE’s manuscript format (see also comment 5)

Comment 7: The statistical analysis performed, although described in the methods section of the manuscript, is not clearly described in relation to the specific groups of data analyzed.

Answer 7: We thank the reviewer for this important comment. We have expanded the methods section with a detailed description of the groups analyzed.

Comment 8: The name of the last Figure reported is Figure 1B instead of Figure 3B, please correct.

In addition, few English mistakes are present in the manuscript, please check and correct.

Answer 8: The figure name was corrected. We have also corrected spelling mistakes and had the manuscript edited by a native speaker with a scientific background which is mentioned in the acknowledgements section.
